# Non-adherence to anti-retroviral therapy among HIV infected adults in Mon State of Myanmar

**DOI:** 10.1186/s12889-017-4309-5

**Published:** 2017-05-05

**Authors:** Win Lei Aye, Apa Puckpinyo, Karl Peltzer

**Affiliations:** 1 Master of Primary Health Care Management Program, ASEAN Institute for Health Development, Mahidol University, Nakhonpathom, Thailand; 2 International Organization for Migration, Kamayut Township, Yangon, Myanmar; 3 ASEAN Institute for Health Development, Mahidol University, Nakhonpathom, Thailand; 4 Department of Research and Innovation, University of Limpopo, Sovenga, South Africa; 5 HIV/STI and TB Research Programme, Human Sciences Research Council, Pretoria, South Africa

**Keywords:** Anti-retroviral therapy, HIV-infected adults, Myanmar, Non-adherence

## Abstract

**Background:**

The provision of Anti-Retroviral Therapy (ART) was started in Myanmar in 2005 in collaboration with the National AIDS Program and the private sector. Successful clinical management of HIV-infected patients is subject to optimal adherence. The aim of the study was to determine the prevalence of adherence to ART and identify factors associated with non-adherence to ART among HIV infected adults registered in a private sector setting in Mon State, Myanmar.

**Methods:**

This cross-sectional study was conducted with adults living with HIV receiving ART at an HIV outpatient clinic between April and May 2016. A total of three hundred People Living with HIV(PLHIV) were interviewed using a pretested and structured questionnaire. The 30 days Visual Analog Scale (VAS) adherence instrument was used to assess the level of adherence. Multivariable logistic regression analysis was used to determine factors associated with non-adherence to ART.

**Results:**

Among 300 patients (male 37.7% and female 62.3%, with a mean age of 41.3 years, standard deviation 8.7), 84% reported ≥95% adherence to ART in the past month. Among 16% of those reporting non-adherence, major reasons for skipping the medication were being busy (23%), being away from home (17.7%) and being forgetful (12.3%). In multivariable logistic rgeression, low behavioural skills on ART adherence (OR = 0.31, 95% CI: 0.10-0.94), tobacco use (OR = 3.22, 95% CI:1.28-8.12), having disclosed their HIV status (OR = 0.07, 95% CI: 0.01-0.69), having a partner who was not on ART (OR = 4.25, 95% CI: 1.70-10.64) and among men, having erectile dysfunction (OR = 15.14, 95% CI: 1.41-162.66) were significant associated with ART non-adherence.

**Conclusion:**

Non-adherence to ART was associated with individual moderating factors and behavioral skills. Priority measures such as addressing risk behaviour and behavioural change communication tailored to individual patients’ lifestyles requires comprehensive interventions to improve adherence.

## Background

Approximately 35 million people were living with the Human Immunodeficiency Virus (HIV) worldwide in 2013, and 1.5 million people had died due to Acquired Immune Deficiency Syndrome (AIDS) related causes [1, 2]. Life-saving HIV treatment, Highly Active Anti-Retroviral Therapy (HAART) is a unique tool in the AIDS response which reduces mortality and moribidity. Declines in morbidity are usuallly measured by occurrence of specific opportunistic infections [3, 4] and Anti-Retroviral Therapy (ART) has lead to approximately 40.2 million life-years gained. Despite this evidence, twenty-two million, or three out of five people living with HIV (PLHIV), are still not able to access ART [5]. As hopes for ending the AIDS epidemic are dependent on providing HIV treatment to all who need it, access to Universal Health Care is also critical [4].

Since 2013, evidence and programmatic experience have led to reductions in HIV related mortality, morbidity and HIV transmission outcomes through earlier initiation of ART. When ART is able to be initiated earlier, the clinical outcome of PLHIV are improved [6]. As ART is scaled up in most of the countries in the world, it is necessary to understand why and how many people drop out of treatment programmes and what factors affect adherence to ART. The concept of an HIV “treatment cascade” is used to describe the entire process of HIV testing, linkage to care, initiation of ART, treatment adherence, and retention in care [4]. A cumulative cross-sectional cascade for HIV treatment and care in Myanmar 2014 reported that out of an estimated 212,000 PLHIV, only 91,000 knew their diagnosis and 85,626 were receiving anti-retroviral therapy [1]. In 2014, amongst ART patients in Myanmar, 9586 received a viral load test with only 8295 achieving suppression of viral load [1]. These reports indicate that only 40% of the PLHIV in Myanmar are prescribed ART and that among these individuals, only 11% received viral load testing and among them, only 87% have suppressed viral loads [1]. Thus, to achieve optimal clinical outcomes of treatment, attention to each step in the treatment cascade is critical.

To achieve a successful treatment outcome, HIV treatment requires more than 95% adherence levels [7, 8]. Adherence research provides strong evidence that an HIV patient with non-adherence to ART has 3.87 times greater mortality rates than an adherent patient on the same treatment [9]. Individual factors contributing to non-adherence may include forgetfulness, being away from home, changes in daily routines, depression or other illness, limited knowledge of treatment benefits and substance use [10]. In a study in Ethiopia conducted among 974 ART patients, 13% were found to have missed one, two or three or more doses when they took their prescribed anti-retroviral drugs (ARVs) during four days recall before interview [11]. In another study in Laos that assessed ART adherence among PLHIV adults receiving free ART at a hospital reported an alarming 40% of the study participants as non-adherent to ART. Patients in that study revealed numerous reasons for their actions, ranging from repoting being very busy to take their treatment to forgetfulness, drinking alcohol and substance use [12]. Moreover, a study in India reported that only 73% of patients on ART took their treatment with at least or over 95% adherence [13]. Furthermore, patients’ perceived erectile dysfunction may be regarded as a relevant problem for HIV-infected persons on anti-retroviral therapy [14] and has been strongly associated with suboptimal ART adherence [15]. According to these studies, maintaining over 95% adherence level among the PLHIV in developing countries is a crucial issue to maintain viral suppression, to minimize HIV related morbidity and mortality and there are many predictors that have been associated with ART non-adherence.

The concept of keeping treatment adherence should start from the earliest involvement in patient care. Retention in care of HIV-infected patients also reflects treatment success at the individual level [16, 17]. Findings of studies in Myanmar for retention in care at private sector ART sites have shown that the reported retention in care at 60 months reached 72% in the 2012 [18] and 74% in the 2014 respectively [19]. Since individual treatment success is associated with retention in care, suboptimal retention is directly associated with negative outcomes on the long term effect on viral suppression and adherence outcomes [20]. Additionally, there are no routine assessments fo ART adherence among PLHIV in Myanmar since most of the settings are resource-limited. Finally, there are very few studies related with ART adherence for PLHIV in Myanmar. Therefore, there is a need to determine the level of adherence among PLHIV in Myanmar. It is important to know the rates of adherence to ART among PLHIV in Myanmar and which factors can be associated with non-adherence to ART. This research study will investigate adherence to ART in Myanmar and identify factors affecting the non-adherence among a sample of PLHIV adults receiving ART in the private sector in Myanmar.

## Methods

### Study setting

The study was carried out at an outpatient ART clinic in the private sector (defined as non-government provider) in Mon State, Myanmar. At the time of the study over 2000 PLHIV were registered at the study site, of which 1423 were on ART. The study site provided all treatment, care and support free of charge and clinical consultations were performed by medical doctors with counselling performed by dedicated nurse counsellors.

### Study design and participants

The cross sectional study was performed from April to May 2016. The study population included HIV-infected adults ≥18 years of age who were confirmed to be HIV positive and currently taking ART for at least one month at the outpatient clinic. The patients who met the inclusion criteria were invited to participate in the research, received an explanation of the purpose of the study and were asked for informed consent. Participants were drawn from the PLHIV ART patients list who consecutively attended the clinic for their follow up to receive ART and/or counselling after asking to participate in the research. To complement the questionnaire, some treatment-related information was extracted through the review of medical records with patients’ informed consent.

### Sampling

The participants were selected by consecutive sampling. The initial sample size was estimated to be 272 with a power of 0.77%. However, we were able to recruit 300 participants who met the inclusion criteria.

### Definition of adherence

Adherence is the extent to which a person is taking the medicine as prescribed by physician and according to medical recommendations, inclusive of timing, dosing and consistency and correctly taking the drugs in terms of right doses, right times and following the dietary recommemdations [21]. Although there is no gold standard for measuring adherence to medication, many measurements of adherence assessment can be used such as self-reports, pill counts, medication event monitoring systems, pharmacy refill tracking and biological markers such as monitoring the level of viral load [22]. Among these measurements, self-report is easy for data collection and can also determine the reasons why patients are non-adherent [22]. Patients assume, however, they can accurately recall their behaviour and are providing sincere answers [23]. The rate of adherence to ART is the number of tablets that the patient has really taken divided by the number of tablets that the patient had to take multiplied by the number one hundred [24].

### Measurement

We used the *Adult AIDS Clinical Trials Group (AACTG)* adherence instrument and 30 days visual analogue scale (VAS) to assess adherence. AACTG is four days recall method for measuring adherence that also assesses reasons of non-adherence [25]. VAS adherence assessment is the Medication Self-report Inventory that can be used to assess adherence to a single antiretroviral medication [26]. It is a self-reported ART measure that is simple to use and has been most extensively used [25]. The subjects are requested to place a mark at the point on a horizontal scale answering to the subject’s best estimation of the proportion of medication doses taken with examples of 0, 50%, and 100% adherence over the past 30 days. Adherence was assessed by self-report adherence methods to categorize study the population in two outcome groups namely “adherence” and “non-adherence”. VAS measures overall adherence for a longer time interval and wide assessment coverage. Adherence refers to the PLHIV took ≥95% of doses taken over the past 30 days. On the other hand, patients with <95% of doses taken over the past 30 days, were defined as non-adherence.

The life *Windows Information-Motivation-Behavioral Skill ART Adherence questionnaire* (LW-IMB-AAQ) was used to assess adherence barriers [27]. Each LW-IMB-AAQ item represents a barrier falling within the I (Information), M (Motivation), or B (Behavioral Skills) constructs. Adherence information was assessed with nine items. Cronbach’s alpha for information in this sample was 0.66. Adherence-related motivation is composed with two factors of personal motivation and social motivation to adhere to one’s regimen. Personal motivation is represented as attitudes on treatment and beliefs about medications. Adherence motivation was assessed with ten items. Cronbach’s alpha for this sub-scale was 0.58. Behavioural skills (adherence ‘self-efficacy’, i.e. patient’s perceived ability to follow medication regimen) was assessed with 14 items. The behavioral skills showed an internal consistency of α = 0.60 for this sample [27]. Response options to theinformation and motivation questions ranged from “1 = strongly disagree to 5 = strongly agree”. Response to items of behavioral skills includes 1 = very hard to 5 = very easy. As scoring instruction, the direction of ‘correct’ was assigned a value of one, while other response options were scored zero in all items of IMB constructs [24]. According to this instructions, for each participant score ‘1’ for each item indicates a response towards adherence and score ‘0’ indicates the item as a barrier. Scores for each domain of IMB (Information-Motivation-Behavioral skills) was split into three levels - low, moderate and high and the percentage of patients in each level will calculate.

*Social support*: Three items were drawn from the Social Support Questionnaire [28] to assess perceived social support. These items are responded to on 4-point scales,1 = completely true, to 4 = completely false, and summed to a score with a range of 3-12. Cronbach’s alpha for this sample was 0.75.

*Internalized AIDS stigma* was assessed with a 7-item AIDS-Related Stigma Scale [29]. Items are responded to from 1 = strongly agree to 4 = strongly disagree. Strongly agree and agree were converted to “1” and strongly disagree and disagree to “0”, scale scores and represent total sum score was range 0-7. Cronbach’s alpha for this stigma index was 0.80 in this sample.

*HIV status disclosure* was assessed with two questions, 1) “Did you disclose your HIV status to your partner?” and 2) “Did you disclose your HIV status to others such as other relatives or friends?”

*Depression*: The Patient Health Questionnaire (PHQ-9) [30] was utilized to screen patients suffering from depression. This 9-item questionnaire asks the patient how emotional problems impact on daily life. The 4-option response format allows scores of 0, 1, 2 and 3 to the response categories of “not at all”, “several days” “more than half the days” and “nearly every day” respectively. The total PHQ-9 score for the nine items ranges from 0 to 27 and scores of 5, 10, 15, and 20 represent cut points for mild, moderate, moderately severe, and severe depression respectively. Cronbach’s alpha for PHQ-9 was 0.76 in this sample. In this study the cut-point of ≥10 scores was used to identify moderate to severe depression.

*Past month alcohol use* was assessed using the Alcohol Use Disorder Identification Test (AUDIT)-C [31]. Cronbach’s alpha of AUDIT-C in this sample was 0.74.

*Tobacco use* was assessed with four questions, adapted from WHO’s Global Adult Tobacco Survey (GATS) [32] by which 1) “*Do you currently smoke any tobacco products, such as cigarettes, cigars or pipes*?” (If, yes: 2) “*Do you currently smoke tobacco products daily*?” 3) “*Do you currently use any smokeless tobacco, such as snuff, chewing tobacco, betel*?” (If, yes: 4) “*Do you currently use smokeless tobacco products daily*?”. Response options were “yes” or “no”.

*Sexual functioning*: An abbreviated 5-items version of the 15-items International Index of Erectile Function (IIEF) to diagnose the presence and severity of erectile dysfunction (ED) [33] was used. The five items selected were based on ability to identify the presence or absence of ED and these are focused on erectile function, e.g., “*How do you rate your confidence that you could get and keep an erection*?” Responses were scored on a five-point ordinal scale where lower values represent poorer sexual function, with a response of 1 the least functional to a response of 5 the most functional. The possible scores for the IIEF-5 ranged from 5 to 25 (one question has scores of 1-5) and ED is classified into four categories: severe (5-7), moderate (8-11), mild to moderate (12-16), mild (17-21), and no ED (22-25) [33].

*Chronic conditions.* Patients were asked if they had been diagnosed by a doctor or other health worker with the following conditions: high cholesterol/high blood lipids, heart disease, stroke, cancer, kidney disease, asthma, arthritis, diabetes (high blood sugar), depression, obesity and other. Patients were classified as having comormidity if they had one or more chronic conditions.

*Sociodemographic factors* assessed included gender, age, marital status, education and occupation.

*Medical file information* included duration of ART, CD4 count, HIV medication and ART regimen.

### Data collection

Face to face interviews were carried out by one researcher with the target population during times participants were waiting to see doctors, nurses or counsellors. The questionnaire was prepared in English and translated into Myanmar language and back translation was also done. Pre-testing of the questionnaire was completed by 30 HIV-infected adults on ART not involved in the study. The questionnaire set has four main parts: section one, background information; section two, adherence indicators; section three, variables under the IMB model; and section four, moderating factors to ART non-adherence. Data collection was done in a private room of the clinic providing confidentiality of the respondent. It took 30-45 min for each participant to complete the interview.

### Data analysis

All data was coded, entered and analyzed by using Statistical Package for the Social Sciences (SPSS) for Windows software application program version 21.0. Data was cleaned before subjected to analysis. All data concerning the variables was analyzed using descriptive statistics. Bivariate analysis were done to find associations between independent variables and the outcome. Only the independent variables that were found to be associated to the outcome at bivariate regression analyses were used in the multiple regression model to examine associations between the outcome of ART non-adherence and independent variables. The statistical significant was set at *p* value <0.05.

## Results

### Socio-demographics

Three hundred patients were recruited for this study. The response rate was 98.7%. Among them, 62.3% were female,54.3% were married and the mean age was 41.3 years (SD = 8.7) (range 18-66). Almost half of the patients (42.7%) had attained primary education level, while 46% had attained secondary school or higher level education. Over half of the respondents (55.3%) were working as casual workers, 20.7% worked in government services or owned a business and 25% were housewives or unemployed (see Table 1).

**Table 1:** Socio-demographic characteristic of respondents (*N* = 300)

Variables	Number	Percent
Gender		
Male	113	37.7
Female	187	62.3
Age in years	Mean ± SD: 41.3 ± 8.7	
< 35	79	26.3
35-45	136	45.3
≥ 46	85	28.3
Marital status		
Single	18	6.0
Married/cohabiting	163	54.3
Separated/divorced/widowed	119	39.7
Education		
No formal education	34	11.3
Primary school	128	42.7
Secondary school	84	28.0
High school	40	13.3
Graduate level	14	4.7
Occupation		
Own business/ Officer	62	20.7
Housewife/Unemployed	75	25.0
Causal workers	163	54.3

*SD* standard deviation

### Health status and behaviour variables

Nearly half of the patients (49%) had been taking ART for one to three years, 42.7% for over three years and 8.3% ART for less than one year. The mean CD4 count was 517.5 (SD = 263.3) cells/mm^3^, with a range of 14 to 1425, and 52.7% of respondents had CD4 cell counts less than 500 cells/mm^3^. Majority of the patients took first line ART drugs (92.3%) and 57% took a one time per day regimen with TDF/ABC + 3TC + EFV. Most of the patients (82.7%) did not have other co-morbid chronic conditions and 17.3% had comorbidity such as heart disease, diabetes, hypertension, asthma, kidney disease, rheumatic disease and high cholesterol. Almost one-fifth (16.7%) engaged in problem drinking, 9.0% smoked daily, 12.3% used smokeless tobacco daily and 7.7% screened positive for moderate to severe depression. The majority (80.3%) had disclosed their HIV status to others (outside the partner, such as other relatives and friends). Among male participants, 13.1% reported mild to moderate ED (see Table 2).

**Table 2:** Health status and behaviour variables (*N* = 300)

Variables	Number	Percent
Duration on ART	Mean ± SD: 4.3 ± 2.1	
< 1 year	25	8.3
1-3 year	147	49.0
> 3 year	128	42.7
Recent CD4 (cells/mm^3^)	Mean ± SD: 517.5 ± 263.3	
< 500	158	52.7
500-1000	122	40.7
> 1000	20	6.7
HIV medication		
ART first line drugs	277	92.3
ART second line drugs	23	7.7
ART Regimen		
TDF/ABC + 3TC + EFV (one time/day)	171	57.0
AZT/ABC + 3TC + NVP/LPV/r (2 times/day)	129	43.0
Co-morbid diseases		
Absence of co-morbidity	248	82.7
Presence of co-morbidity	52	17.3
Past month alcohol use	50	16.7
Smokes tobacco daily	27	9.0
Smokeless tobacco use daily	37	12.3
Disclosure of HIV status to others	241	80.3
Moderate to severe depressive symptoms	23	7.7
Internalized stigma (Range:0-7)	Mean ± SD	2.7 ± 2.3
No stigmatization	67	22.3
Low stigmatization	132	44.0
High stigmatization	101	33.7
Social support (Range 3-12)	Mean ± SD	9.7 ± 2.0
Less	194	64.7
High	106	35.3
Sex functioning among male respondent (*n* = 107) (Range 5-25)	Mean ± SD: 18.9 ± 2.7	
No erectile dysfunction (ED)	49	45.8
Mild ED	44	41.1
Mild to moderate ED	14	13.1

*TDF* Tenofovir, *ABC* Abacavir, *3TC* Lamivudine, *EFV* Efavirenz, *AZT* Zidovudine, *NVP* Nevirapine, *LPV/r* Lopinavir/ritonavir, *SD* standard deviation

### Descriptive statistics on the information-motivation-Behavioural skills ART adherence model

The mean score of the IMB ART adherence information was 4.0 (SD = 2.2), with a range of 0-9 scores, the mean score of the IMB ART adherence motivation was 8.7 (SD = 1.5), with a range of 0-10 scores, and the mean score of the IMB ART adherence behavioural skills was 11.7 (SD = 1.4), with a range of 0-14 scores (see Table 3).

**Table 3:** Information-motivation-behavioural skills model (*N* = 300)

Variables	N or Mean	% or SD
IMB adherence information^a^	Mean ± SD	4.0 ± 2.2
Low	122	40.7
Moderate	90	30.0
High	88	29.3
IMB adherence motivation^a^	Mean ± SD	8.7 ± 1.5
Low	54	18.0
Moderate	118	39.3
High	128	42.7
IMB behavioral skills^a^	Mean ± SD	11.7 ± 1.4
Low	111	37.0
Moderate	91	30.3
High	98	32.7

*SD* standard deviation

^a^higher score = higher adherence information, motivation and behavioural skills

### Descriptives of ART adherence

The assessment of ART adherence is summarized in Table 4. The categories of adherence to ART of the respondents were measured by asking them if they missed taking any medication during the past four days and/or past 30 days periods. The mean percentage of adherence of 30-days VAS score was 97.3 (SD = 5.0), with a range of 80.0-100.0. In order to examine the adherence during the past 30 days, two groups were created using the score of 95% to benchmark optimal adherence. Among the respondents, 84% (*n* = 252) reported an adherence scores ≥95% and were allocated to the adherence group and participants with adherence scores <95% were allocated to a non-adherence group, representing 16% of the respondents (*n* = 48). For the four day period prior to the questionnaire administration, patients missing at least one dose of their medication during that period were allocated to the dose non-adherence group and patients missing no dose were allocated to the dose adherence group. It was seen that twenty nine patients (9.7%) reported having missed at least one dose appointment during the past four days while most of them reported having no skipped dose (90.3%). All the respondents who had missed their dose were asked the reasons for this behaviour. As shown in Table 4, the major reason given for non-compliance were being too busy with other things (23%), away from home (17.7%), or forgetfulness (12.3%).

**Table 4:** ART non-adherence

Variable	Number	Percent
30 days VAS		
≥ 95% adherence	252	84.0
< 95% adherence	48	16.0
Self-report 4 days recall dose adherence		
Adherence	271	90.3
Non-adherence	29	9.7
Reasons for missed medication (*n* = 130)		
Busy	69	23.0
Away home	53	17.7
Forget	37	12.3
Change in daily routine	23	7.7
Asleep	15	5.0
Sick	13	4.3
Not want others to notice	8	2.7
Side effects	6	2.0
Too many pills	4	1.3
Problems with taking pill	4	1.3
Depressed	3	1.0
Feel toxic	2	0.7
Ran out of pills	2	0.7

*VAS* Visual Analog Scale

### Associations with ART non-adherence in the past 30 days

The result of bivariate analysis revealed a statistically significant association with ART non-adherence in patients working as own business and government officers compared to those who are working as housewife and unemployed or causal workers (*p* = 0.039). Patients who had moderate scores on ART information were more likely to be non-adherence to ART (*p* = 0.005) than patients with low scores on ART information. PLHIV who had higher scores on behavioural skills to take the medication reported better adherence than those who had low and moderate scores in behavioural skills (*p* = 0.048). Problem drinking, smoking daily and daily smokeless tobacco use were found to be associated with ART non-adherence (*p* = 0.038, 0.007 and 0.005, respectively). The patients who had disclosed their HIV status to others were more likely to be non-adherent to ART than those who had not disclosed (*p* = 0.018). The patients whose partner was not on ART were more likely non-adherent than patients who had a partner on ART (*p* = 0.002). Men who had mild to moderate ED had a higher odds to non-adherence than those without or mild ED (*p* = 0.008).

In multivariable logistic regression analysis, poor IMB ART adherence behavioural skills, daily smokeless tobacco use, having disclosed their HIV status to others, having a partner who was not on ART, and among men mild to moderate ED was associated with ART non-adherence. Due to few cases with ED, confidence intervals are wide (see Table 5).

**Table 5:** Prevalence of and associations with ART non-adherence in the past 30 days

Variable	n	%	COR (95% CIs)	*P*-value	AOR (95% CIs)	*P*-value
Gender						
Female	25	13.4	1 (Reference)			
Male	23	20.4	1.66 (0.89-3.09)	0.112		
Age (years)						
< 35	16	20.3	1 (Reference)			
36-45	20	14.7	0.68 (0.33-1.40)	0.295		
46 and above	12	14.1	0.65 (0.29-1.47)	0.299		
Marital status						
Single	5	27.8	1 (Reference)			
Married/cohabiting	28	17.2	0.54 (0.18-1.63)	0.275	---	
Separated/divorced/ widowed	15	12.6	0.38 (0.12-1.20)	0.099		
Education						
Primary and less	24	14.8	1 (Reference)			
Secondary and above	24	17.4	1.21 (0.65-2.25)	0.544	---	
Occupation						
Housewife/student/unemployed	8	10.7	1 (Reference)			
Own business/officers	15	24.2	2.67 (1.05-6.81)	0.039	1.43 (0.54-3.82)	0.474
Casual worker	25	15.3	1.52 (0.65-3.54)	0.335	1.34 (0.44-4.98)	0.789
Duration on ART						
> 3 years	26	20.3	1 (Reference)			
1-3 years	18	12.2	0.55 (0.28-1.05)	0.071	---	
< 1 year	4	16.0	0.75 (0.24-2.37)	0.620		
Recent CD4 count						
≥ 500-1000	23	16.2	1 (Reference)			
< 500	25	15.8	0.97 (0.52-1.80)	0.930	---	
HIV medication						
ART first line drugs	45	16.2	1 (Reference)			
ART second line drugs	3	13.0	0.77 (0.22-2.71)	0.688	---	
ART regimen						
TDF/ABC + 3TC + EFV	23	13.5	1 (Reference)			
AZT/ABC + 3TC + NVP/LPV/r	25	19.4	1.55 (0.83-2.87)	0.167	---	
Diagnosis of chronic disease						
No chronic disease	42	16.9	1 (Reference)			
Has chronic disease	6	11.5	1.56 (0.63-3.90)	0.338	---	
IMB adherence information						
Low	13	10.7	1 (Reference)		1 (Reference)	
Moderate	23	25.6	2.87 (1.37-6.06)	0.005	1.96 (0.67-5.74)	0.222
High	12	13.6	1.32 (0.57-3.06)	0.511	0.58 (0.17-1.96)	0.380
IMB adherence motivation						
Low	7	13.0	1 (Reference)			
Moderate	18	15.3	1.21 (0.47-3.09)	0.693	---	
High	23	18.0	1.47 (0.59-3.67)	0.408		
IMB adherence behavioural skills						
Low	24	21.6	1 (Reference)		1 (Reference)	
Moderate	13	14.3	0.60 (0.29-1.27)	0.183	0.37 (0.13-1.07)	0.066
High	11	11.2	0.45 (0.21-0.99)	0.048	0.31 (0.10-0.94)	0.039
Past month alcohol use (base = no)	13	26.0	2.16 (1.04-4.46)	0.038	0.80 (0.25-2.55)	0.702
Smokes tobacco daily (base = no)	16	28.1	2.57 (1.29-5.12)	0.007	2.38 (0.85-6.65)	0.098
Smokeless tobacco use daily (base = no)	23	25.3	2.49 (1.32-4.68)	0.005	3.22 (1.28-8.12)	0.013
Non-disclosure of HIV status to others (base = yes)	3	5.1	0.23 (0.07-0.78)	0.018	0.07 (0.01-0.69)	0.022
Low social support (base = high)	32	16.5	1.11 (0.58-2.14)	0.752	---	
Internalized stigma						
No stigmatization	8	11.9	1 (Reference)			
Low stigmatization	26	19.7	1.80 (0.77-4.25)	0.174	---	
High stigmatization	14	13.9	1.19 (0.47-3.01)	0.718		
Partner who is not on ART	19	23.2	3.34 (1.58-7.07)	0.002	4.25 (1.70-10.64)	0.002
Moderate to severe depression symptoms (base = no)	4	17.4	1.12 (0.36-3.44)	0.850	---	
Erectile dysfunction among male respondents (base = no)	15	25.9	6.00 (1.61-22.42)	0.008	15.14 (1.41-162.66)	0.025

*IMB* Information, Motivation and Behavioural skills, *COR* Crude Odds Ratio, *AOR* Adjusted Odd Ratio, *CIs* = Confident Intervals

## Discussion

The prevalence of adherence to ART was 84% measured by the 30 days VAS adherence measure in this study. Among the 300 PLHIV in the study, 16% reported a failure to adhere to ART during the past month. The proportion of adherence was found to be different than the results of the most recent ART adherence study in Myanmar which reported 76.2% adherence in 2016 [34]. The discrepancy might be due to adherence measurement tools (pill identification test versus VAS within 30 days). There were also differences in the socio-economic status of the two studies in terms of the target population and the studies were conducted in different treatment settings as well. The aforementioned study was undertaken at a public sector site whilst this current study was conducted in the private sector. A possible reason for the different prevalence of ART adherence between the two settings include a better patient provider ratio and the affiliated consultation time for clients in a private sector setting with relatively better resources than in the public sector [19]. In fact, in this study, the level of adherence was comparable with other studies across the Southeast Asian region where ART adherence varied widely from 60% to 77% [12, 35, 36].

In this study, socio-demographic characteristics of the respondents were not associated with ART non-adherence. Furthermore, previous research has also shown no associaation with gender and non-adherence outcomes, similar to our study [37]. In spite of employment being a contributing factor as financial barrier to patients according to the literature [38], the current study did not reflect this finding since the clinic provided all care free of charge. For adherence related to IMB model, the present study shows that behavioural skills was associated with self-report VAS adherence in both bivariate and multivariate analysis which is consistent with findings from other studies [39, 40]. Findings are logical in the sense that if patients have more self-efficacy themselves, they are better equipped to reach the goal of optimal adherence.

For behaviour variables of moderating factors, tobacco users were significantly associated with non-adherence in our study which is consistent with previous Myanmar study findings where non-smokers were more likely to adhere to ART [34]. We found an increased risk of non-adherence among individuals who had disclosed their HIV status to others and this finding was inconsistent with other studies [41]. Surprisingly, having disclosed to others was associated with non-adherence to treatment. This might have happened because in this study, being a cross-sectional one, could not provide materialistic relations between the two variables. Having a partner on ART was a strong predictor of adherence to ART. This finding supports the role of HIV service providers and counsellors to emphasize couple counselling and encourage HIV testing to discordant couples. In this study, male respondents were assessed regarding sexual functioning during the past six months since previous literature reported increasing occurrence of sexual dysfunction among patients on ART [37]. Among 107 respondents men (as 6 men denied to answer to IIEF questionnaire), 54.2% reported some degree of dysfunction in sexual activities, therefore more than half of patients reporting some degree of erectile dysfunction, similar to other studies [42]. The finding in the present study of an association between sexual dysfunction and non-adherence to ART was confirmed, as found in several previous studies [14, 43].

### Study limitations

This cross-sectional study has several limitations. The ART adherence levels may be overestimated and highly subjective due to self-reporting, and may be prone to recall bias. In order to minimize this bias, however, the answers were reconfirmed and the clients were provided with enough time to remember event. Also, although all participants were invited to participate, the study may have a selection bias, because participants were patients on ART treatment who came to the clinic at the time of data collection. This excluded patients who were not retained in the clinic and also patients who missed clinical appointments during the period. Moreover, the cross-sectional nature of the study design limited the ability of the analysis to determine the direction of the causation. The data were collected from only one private sector clinic of one region in Myanmar, so it is not generalizable to other regions and also the public sector sites.

## Conclusions

The proportion of adherence in this current study was relatively high compared with other studies conducted in Southeast Asia. Around eight out of ten patients who were taking ART reported good adherence to ART. However, suboptimal adherence within some certain subgroups is still a challenging issue. Innovative approaches including daily mobile phone reminders and provision and use of memory aids could be trialled for improving adherence for patients with identified risk factors. Certainly, the present study suggests that the IMB model represented adherence within this sample, as behavioural skills were critical and intervention approaches should be tailored to the targeted population. An important recommendation of this study is that care and support for the people who reported substance use should be included a comprehensive core package of HIV management including risk assessment and behaviour change communication tailored to individual patient’s lifestyles.

## Abbreviations

AACTG: Adult AIDS Clinical Trials Group
AIDS: Acquired immunodeficiency syndrome
ART: Anti-Retroviral Therapy
ARV: Anti-retroviral drugs
AUDIT: Alcohol Use Disorder Identification Test
ED: Erectile Dysfunction
GFATM: Global Fund for AIDS, TB and Malaria
HAART: Highly Active Anti-Retroviral Therapy
HIV: Human immunodeficiency virus
IIEF: International Index of Erectile Function
IMB model: Information, Motivation and Behavioural Skills Model
LW-IMB-AAQ: Information-Motivation-Behavioral Skill ART Adherence questionnaire
PLHIV: People living with HIV
VAS: Visual Analog Scale
